# Cutaneous Angiosarcoma: The Possibility of New Treatment Options Especially for Patients with Large Primary Tumor

**DOI:** 10.3389/fonc.2018.00046

**Published:** 2018-03-02

**Authors:** Yasuhiro Fujisawa, Koji Yoshino, Taku Fujimura, Yoshiyuki Nakamura, Naoko Okiyama, Yosuke Ishitsuka, Rei Watanabe, Manabu Fujimoto

**Affiliations:** ^1^Dermatology, University of Tsukuba, Tsukuba, Ibaraki, Japan; ^2^Dermatology, Tokyo Metropolitan Komagome Hospital, Tokyo, Japan; ^3^Dermatology, Tohoku University, Sendai, Japan

**Keywords:** cutaneous angiosarcoma, concurrent chemoradiotherapy, maintenance chemotherapy, adjuvant chemotherapy, taxanes, eribulin, pazopanib, angiosarcoma of the scalp

## Abstract

The most widely accepted treatment for cutaneous angiosarcoma (CAS) is wide local excision and postoperative radiation to decrease the risk of recurrence. Positive surgical margins and large tumors (T2, >5 cm) are known to be associated with poor prognosis. Moreover, T2 tumors are known to be associated with positive surgical margins. According to previous reports, the majority of CAS patients in Japan had T2 tumors, whereas less than half of the patients in the studies from western countries did so. Consequently, the reported 5-year overall survival of Japanese CAS patients without distant metastasis was only 9%, lower than that for stage-IV melanoma. For patients with T2 tumors, management of subclinical metastasis should be considered when planning the initial treatment. Several attempts to control subclinical metastasis have been reported, such as using adjuvant/neoadjuvant chemotherapy in addition to conventional surgery plus radiation. Unfortunately, those attempts did not show any clinical benefit. Besides surgery, new chemotherapeutic approaches for advanced CAS have been introduced in the past couple of decades, such as paclitaxel and docetaxel. We proposed the use of chemoradiotherapy (CRT) using taxanes instead of surgery plus radiation for patients with T2 tumors without distant metastasis and showed a high response ratio with prolonged survival. However, this prolonged survival was seen only in patients who received maintenance chemotherapy after CRT, indicating that continuous chemotherapy is mandatory to control subclinical residual tumors. With the recent development of targeted drugs for cancer, many potential drugs for CAS are now available. Given that CAS expresses a high level of vascular endothelial growth factor (VEGF) receptor, drugs that target VEGF signaling pathways such as anti-VEGF monoclonal antibody and tyrosine kinase inhibitors are also promising, and several successful treatments have been reported. Besides targeted drugs, several new cytotoxic anticancer drugs such as eribulin or trabectedin have also been shown to be effective for advanced sarcoma. However, most of the clinical trials did not include a sufficient number of CAS patients. Therefore, clinical trials focusing only on CAS should be performed to evaluate the effectiveness of these new drugs.

## Background

According to the Surveillance, Epidemiology, and End Results Program database, the number of patients with sarcoma recorded between 2010 and 2014 was only 1/100 of the number of patients with carcinoma in the same period. Moreover, angiosarcoma accounts for only 1% of all sarcomas, so patients with angiosarcoma constitute only 1 in 10,000 of all patients with malignant neoplasms (1–3). Although the incidence of angiosarcoma has increased in the past couple of decades, it is around 0.5 per 1,000,000 persons, or fewer than 200 new patients, per year in the United States (3). Owing to this rarity, most previous publications have been case reports or small case series, making it difficult to interpret the results because of the selection bias and small number of patients included in those studies. Furthermore, because of this rarity, no randomized phase-3 study has been conducted, especially for angiosarcoma, and consequently, no clinical trial-proven standardized treatment has thus far been established. Although complete removal of the tumor was believed to be essential, as it is for other sarcomas (4, 5), some reports have suggested that wide-margin surgery will not deliver favorable results (6, 7). In this review, we will summarize the clinical features and current treatments of angiosarcoma and discuss the possibility of new therapeutic options for this rare disease.

## Clinical Presentation

Angiosarcoma develops in various soft tissues and organs, but the most commonly affected site is the skin [cutaneous angiosarcoma (CAS)] (8–10). According to an analysis of 434 cases of CAS, 62.1% of them developed in the head and neck, 24.4% in the trunk, 10.6% in the extremities, and 2.7% in other locations (11). CAS commonly occurs in the scalp and typically presents as an enlarging bruise-like purpura in the head and neck region and may be associated with ulceration and/or a tumor. Sometimes patients develop a thick blood crust. These head and neck CAS commonly develops in older men (12–14), whereas the secondary CAS, lymphedema-associated CAS [so-called Stewart-Treves syndrome (15)] and radiation-associated CAS (11, 16), usually develops within the lymphedema site and irradiated field >5 years after the surgery and radiation, respectively (12, 16).

Stewart-Treves syndrome was originally reported as lymphedema that developed after radical mastectomy and lymph node dissection (15), but in the past 15 years, we have never encountered Stewart-Treves syndrome that developed after the surgery for mammary carcinoma. Instead, in the same period, we experienced three cases of Stewart-Treves syndrome that developed in the lower limb after treatment for uterine carcinoma (17). This may be explained by the fact that the number of patients receiving conservative treatment for mammary carcinoma has increased, and as a consequence, the prevalence of Stewart-Treves syndrome in the upper extremity has decreased (18). On the other hand, the occurrence of radiation-induced CAS in the breast is likely to increase given that the prognosis for mammary carcinoma is gradually improving and radiation is more often used to treat (16).

While the incidence of Stewart-Treves syndrome is not well known, it has been reported to be about 1/10 to 1/20 of all CAS (19–22). Similarly, the cumulative incidence of radiation-associated CAS 15 years after radiotherapy for breast carcinoma was reported to be 0.9 per 1,000 patients (23), meaning less than 1 occurrence per 10,000 irradiated patients per year. In this review, considering its rarity and etiological difference, we will focus mainly on primary CAS, the narrow sense of CAS (24).

Distant metastasis could occur within a month of primary surgery, but typically it occurs on average after a year (4, 5). The most common site of metastasis is the lung, followed by the lymph nodes, bone, and liver (4, 5, 25). Interestingly, lung metastasis often presents as pneumothorax, which may require urgent medication (26, 27).

## Diagnosis and Staging

Patients with typical presenting symptoms can be diagnosed clinically, but the precise pathological diagnosis should be performed by an expert pathologist. The histologic features of angiosarcoma can vary between patients and even within the same patient. When the tissue specimens are taken from well-differentiated areas, the tumor cells usually form vessel-like structures and may be difficult to differentiate from normal vessels. However, the tumor vessels tend to form independent or separate networks with anastomoses (28). Other features such as cellular atypia, mitoses, and formation of multilayer endothelium can be helpful for diagnosis. On the other hand, in poorly differentiated areas, the tumor cells show sheet-like growth with hemorrhage and necrosis, which have fewer features than do vascular tumors. In such cases, positive staining for endothelial markers such as CD31, CD34, von Willbrand factor, and vascular endothelial growth factor (VEGF) are useful (29). Also, lymphatic endothelial markers such as D2-40 are positive for most superficial angiosarcomas (28).

The staging of CAS is based on the TNM staging system of the American Joint Committee on Cancer (AJCC) (Table 1). The tumor grade based on the pathologic features is included in the staging. In brief, localized disease is classified as stage I or II; nodal spread or T2 tumor with histologic grade 3, as stage III; and distant disease, as stage IV. However, because there is no standardized treatment algorithm for each stage, staging of CAS has little clinical benefit in the treatment decision.

**Table 1:** AJCC TNM staging system for soft tissue sarcoma.

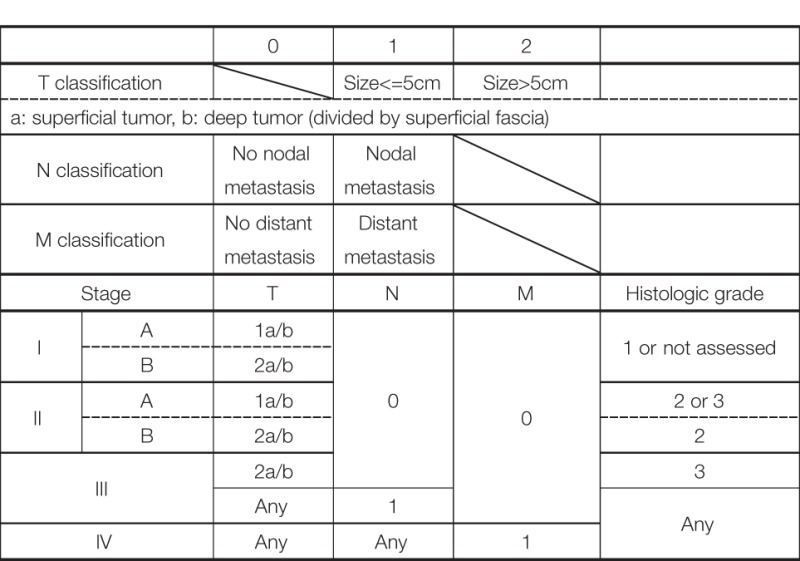

*Histologic grading is defined as follows: (1) Differentiation: score from 1 to 3, (2) Mitotic count: score from 1 to 3, and (3) Tumor necrosis: score from 0 to 2*.

*Sum (1) to (3) and determine grade as follows*.

*Gx: not assessed*.

*G1: total score of 2 or 3*.

*G2: total score of 4 or 5*.

*G3: total score of >5*.

## Prognosis and Factors Associated with Survival

Generally, soft-tissue sarcomas have a 50–60% survival rate (30), whereas the 5-year survival rate for angiosarcoma is <40% (12, 25, 31, 32). Several factors are reportedly associated with poor survival: older age (25, 32), worse performance status (33, 34), larger tumor size (5, 8, 20, 32, 35–40), positive margin status (31, 32, 38, 41, 42), higher histologic type or grade (32, 37, 41, 43, 44), scalp as the primary location (5, 36, 45), deeper location of the tumor (20, 31), and presence of distant metastasis (33, 38, 41, 46). On the other hand, the following factors were associated with favorable prognosis: surgery (20, 34), multimodal therapy (5, 39, 41) and postoperative radiotherapy (34, 36, 41, 43, 47, 48). The studies that included more than 50 patients with CAS only are summarized in Table 2. According to these five studies, tumor size seems to be a consistently poor prognostic factor; indeed, patients with tumors larger than 10 cm all died of the disease (35, 36).

**Table 2:** Reported factors associated with poor survival determined by studies with >50 patients in CAS.

	*N*	Age	Tumor size	Pathological feature	Margin	Location	Others
Albores-Saavedra et al. (11)	434	>50	N.S.			Head and neckDeeper location	Lymph node metastasisDistant metastasis

Perez et al. (40)	88	N.S.	>5 cm		N.S.		

Holden et al. (36)	72	N.S.	>5 cm >10 cm	N.S.		N.S.	

Guadagnolo et al. (5)	70		>5 cm Satellitosis		N.S.		Surgery alone

Patel et al. (25)	55	>70	N.S.	N.S.	N.S.		Without multimodality or radiation therapy

*N.S., not significant; PS, performance status*.

A study by Sinnamon et al. (32) of 821 angiosarcomas included 211 cases of primary CAS in the head and neck. In their cohort, all cases of metastatic disease were excluded and all the patients received surgical treatment. They scored the following factors and classified the risk from low (total score 0–1), intermediate (total score 2–3), and high (total score 4–7): age > 70 as 1, black ethnicity as 1, histologic tumor grade 3 as 1, tumor size 3–7 cm as 1, tumor size larger than 7 cm as 2, microscopic residual tumor as 1, and macroscopic residual tumor as 2. By using this model, patients at high risk had a median overall survival of only 1.6 years with a hazard ratio of 5.65 when compared with patients at low risk. This result clearly indicates that these factors strongly correlate with poor survival.

Reports from Japan and from western countries showed differences in survival. In the study from Japan of 260 cases of CAS, the 5-year overall survival among patients who could receive surgery was <20% (49) (median overall survival: < 20 months), whereas in the studies from western countries, it was 31–51% (5, 11, 25, 31, 40). CAS patients in Japan had equivalent survival to the “high risk” group reported by Sinnamon et al. (32), with a median overall survival of 1.6 years. This difference might be explained by the fact that the tumor size in Japanese patients is generally large: in the study of 260 CAS cases, 44% of the patients had tumors of at least 10 cm (originally, described as tumors larger than 100 cm^2^) (49), whereas tumors larger than 5 cm (T2) constituted only 18–38% of the patients in the studies from western countries (5, 11, 25, 40). Our multicenter study, which included only Japanese patients, was also T2 dominant: only 3 of 28 patients (11%) had a T1 tumor (19). In the meta-analysis by Hwang et al., which included 128 cases from seven studies (50), the median overall survival in the T1 group was significantly longer than that in the T2 group (31.4 months and 17.3 months, respectively: *P* < 0.001). Collectively, Japanese CAS patients have larger primary tumors than do CAS patients in western countries, and consequently, the survival of Japanese CAS patients is shorter.

## Treatment

### Current Treatment Options

#### Surgery

Radical surgery with no residual tumor cell on the margin (R0 resection) is generally the primary goal of sarcoma treatment. In every review or set of guidelines, surgery with R0 resection is recommended as the goal of CAS treatment (28). In a systematic review by Shin et al. (51), absence of surgery was shown to correlate with poor survival; Trofymenko et al. (52) reported similar result in a study using 764 cases of CAS extracted from the National Cancer Database in the United States. Therefore, there is little doubt that surgery is one of the best choice for the management of CAS.

Although no standardized treatment recommendation has been established, a margin of less than 1 cm is associated with poor survival (49). The depth of the resection has not been well discussed, but generally if the tumor does not extend into the deep fascia, a resection layer including the deep fascia is adequate. If the tumor directly invades into the deep fascia, removal of the underlying structures, e.g., the periosteum or even the outer shell of the skull, is required to obtain R0 resection.

Unfortunately, it is common to see positive microscopic (R1) or macroscopic (R2) margins even after a wide surgical margin from the visible tumor border has been obtained (4, 8, 31, 36, 41). Pawlik et al. (4) reported that in their series of 29 patients, 18 (62.1%) had an initial diagnosis of T1 (<5 cm) tumor, but 11 of those tumors turned out to be T2 (>5 cm) after surgical pathology evaluation of the resected tumor. The clinical margin of the tumor in CAS is difficult to determine because it often develops as a multifocal tumor and presents as a skip lesion. Moreover, when CAS develops near important structures such as the eye, surgical removal with an adequate margin is impossible. As a consequence, the rate of local recurrence after treatment is high reportedly ranging from 26 to 100% (5, 9, 25, 41). Lahat et al. (53) reported 32 of 44 cases of locally recurrent angiosarcoma treated with surgery, 70% of which achieved complete removal of the recurrent tumor, with a 5-year overall survival of 44%.

To reduce local recurrence, postoperative radiotherapy covering a wide area with a >50 Gy dose has been reported by several studies to be effective not only for local control but also for overall survival (4, 5). Currently, wide local excision followed by radiation is the most accepted treatment for CAS (28, 54, 55); however, despite such mutilating multimodal treatment, survival of patients, especially of those with large tumors, is still unsatisfactory (19, 32).

Other than radical surgery, palliative surgery might have role in patients with large tumors to reduce the tumor load. Some reports suggested the use of minimal surgery as part of the management of CAS (6, 56), such as for those cases with a diffuse lesion pattern involving vital structures, recurrent disease, or metastasis.

#### Chemotherapy

The chemotherapeutic options currently available for angiosarcoma are listed in Table 3. Chemotherapy using anthracyclines alone or in combination with ifosfamide have been used for unresectable and metastatic angiosarcoma (35, 57, 58). However, anthracyclines have cardiac toxicity which make it difficult to apply in older patients. Taxanes, which inhibit tubulin elongation, were introduced in the 1990s as a novel cytotoxic drug and have become accepted as standardized treatment options in various kinds of cancers such as those of the breast (59), lung (60), stomach (61), and uterus (62), because of their high efficacy. Although several clinical studies have shown that taxanes are of little benefit for sarcomas (63, 64), the angiosarcomas included in those clinical studies showed antitumor activity (64). Taxanes not only have a direct antitumor effect but also have been shown to exert an antiangiogenic effect (65, 66), which is thought to be suitable for the treatment of vascular tumors. Indeed, taxanes were shown to be effective for the treatment of Kaposi sarcoma (67, 68).

**Table 3:** Chemotherapy options and their effect for angiosarcoma.

Agent	Patients	*N*	Response/median survival (months)
Anthracyclines	Pooled analysis of 11 clinical trials for angiosarcoma from all sites Young et al. (58)	108	Response ratio: 25% for all sites PFS: 4.9, OS: 9.9

Paclitaxel	Retrospective review of angiosarcoma from all sites Italiano et al. (71)	34	Response ratio: 29.5% for all sites
		68	Response ratio: 53% for all sites Response ratio: 78% for CAS

	ANGIOTAX study: phase-2 study including angiosarcoma from all sites Penel et al. (70)	30	Response ratio: 18% for all sites Response ratio: 89% for CAS

	ANGIOTAX plus study: phase-2 study comparing paclitaxel with/without bevacizumab from all sites (showing paclitaxel arm only) Ray-Coquard et al. (74)	24	Response ratio: 45.8% for all sites PFS: 6.6, OS: 19.5

	Retrospective study for head/neck CAS Fata et al. (69)	9	Response ratio: 89% for CAS

Docetaxel	Retrospective study for CAS Nagano et al. (101)	9	Response ratio: 67% for CAS

Gemcitabine	Retrospective study with angiosarcoma from all sites Stacchiotti et al. (76)	25	Response ratio: 64% for all sites PFS: 7, OS: 17

*PFS, progression-free survival; OS, overall survival; CAS, cutaneous angiosarcoma*.

In 1999, Fata et al. (69) achieved a response ratio of 89% by using paclitaxel monotherapy for the treatment of head and neck CAS. Later, Penel et al. (70) conducted the first phase-2 trial for metastatic or locally advanced angiosarcoma, which included 30 patients treated with paclitaxel. In that clinical trial, the progression-free survival rate after 4 months was 45%, and the median overall survival was 8 months. Considering that the patients with distant metastasis consisted of 74% of the study population and 36% of them had had previous systemic chemotherapy, this result was encouraging. Italiano et al. (71) showed, albeit in a retrospective study, that paclitaxel achieved an equivalent outcome to that of anthracyclines in the treatment of advanced angiosarcoma despite the patients treated with paclitaxel being a decade older than those treated with anthracyclines (67.4 and 57.4 years old, respectively). Collectively, taxanes can achieve a similar level of antitumor effect to that achieved by anthracyclines, but with less toxicity, and therefore, a recent report (72) suggested using taxanes as the first-line treatment for CAS with unresectable or distant disease. Indeed, we reported (17, 73) successful treatment results using taxanes as the first-line therapy for primary CAS.

Because both taxanes have been reported to be effective, the decision about which taxane to use as the first-line might be difficult. In this review, we recommend paclitaxel as the first-line treatment since paclitaxel has been evaluated in different phase-2 studies (70, 74), whereas docetaxel has not yet been evaluated in a prospective study. However, docetaxel still has a role as a second-line therapy in patients refractory to paclitaxel (75).

Gemcitabine has been reported to be effective for sarcomas both as a single agent (76, 77) and in combination with docetaxel (78, 79). Several case series (77, 80) have been reported in which gemcitabine for the treatment of angiosarcoma was used with favorable outcomes. Moreover, albeit in a study based on a retrospective pooled analysis (76), gemcitabine showed an overall response rate of up to 68% for angiosarcoma (76). If this agent is used as monotherapy, the toxicity profile is better than that of anthracyclines but still has a significant incidence of bone marrow suppression.

#### Radiation

Radiation is usually delivered after surgery for better local control (28, 54, 55). However, dismal outcome have been reported when radiation was used as monotherapy (5, 38, 43). Therefore, radiation monotherapy is generally used for palliation, not for curative intent because of frequent recurrence, as high as 100% in previous studies (25, 36, 42, 43). On the other hand, Ogawa et al. (34) reported that in their cohort of 25 patients who received radiation monotherapy with curative intent, 11 of the 14 patients (79%) who received >70Gy achieved local control, whereas only 3 of the 11 patients (27%) who received <70 Gy did. A study by Scott et al. (81) of 41 patients treated with radiation recommended at least 60–65 Gy for the postoperative tumor bed and 70–75 Gy for patients who receive radiation monotherapy. Others (82) suggested that improved delivery of radiation might achieve higher efficacy. Since no prospective study has been conducted to evaluate the role of radiation as the first-line therapy, radiation monotherapy is still difficult to use as curative intent therapy for primary disease. We will discuss combination radiation and chemotherapy in the next section.

### New Treatment Options

#### Chemoradiotherapy (CRT)

The use of chemotherapy and radiation (CRT) concurrently or concomitantly is one of the standardized treatment methods for several cancers: esophageal (83), head and neck (84), rectal (85), and cervical (86). Chemotherapeutic agents such as 5-fluorouracil (84, 85), cisplatin (87), gemcitabine (88), and taxanes (89, 90) are expected to act not only as cytotoxic but also as radiosensitizing agents. Therefore, CRT may sometimes cause higher toxicity than does monotherapy but can be justified by its high antitumor effect, and in most cases, such side effects are manageable. Besides, although many cancer treatments introduced CRT as one of the key treatments, it was an uncommon method among cutaneous malignancies. In such a situation, we started to use cisplatin and 5-fluorouracil concurrently with radiation for the management of unresectable/metastatic cutaneous squamous cell carcinoma with the same protocol used in the head and neck and reported successful treatment results (91–93).

As described previously, a Japanese retrospective study of CAS (49) revealed that the median overall survival of patients with non-metastatic localized CAS who received surgery was less than 20 months, but this finding was not surprising because we have reported a similar dismal outcome (13.5 months) (19). We suspected that increased expression of VEGF during the wound healing process (94) caused by mutilating surgery might cause progression of residual angiosarcoma because angiosarcoma has been reported to express a VEGF receptor (95–97). As discussed in the previous section, tumor size is the most common factor for poor prognosis, which is commonly related to a positive surgical margin. Therefore, it is convincing to consider that such subclinical residual tumors could be expanded by VEGF released by surgery.

In such a situation, a retrospective study (47) of use of chemotherapy (anthracyclines) and radiation for five head and neck CAS (four scalp and one lip, three of them with high-grade tumors) was reported and achieved a median overall survival of 27.0 months, which was better than the reported median survival of face and scalp CAS (<20 months) (6, 36, 45). However, there was a concern related to use of anthracyclines for older CAS patients for whom the drug might not be tolerable. On the other hand, taxanes have a better toxicity profile, and therefore, we expected that older CAS patients could tolerate it. Moreover, taxanes are known as radiosensitizers (89, 90), and therefore, possibly an ideal agent for CRT for the treatment of CAS.

**Figure 1:**
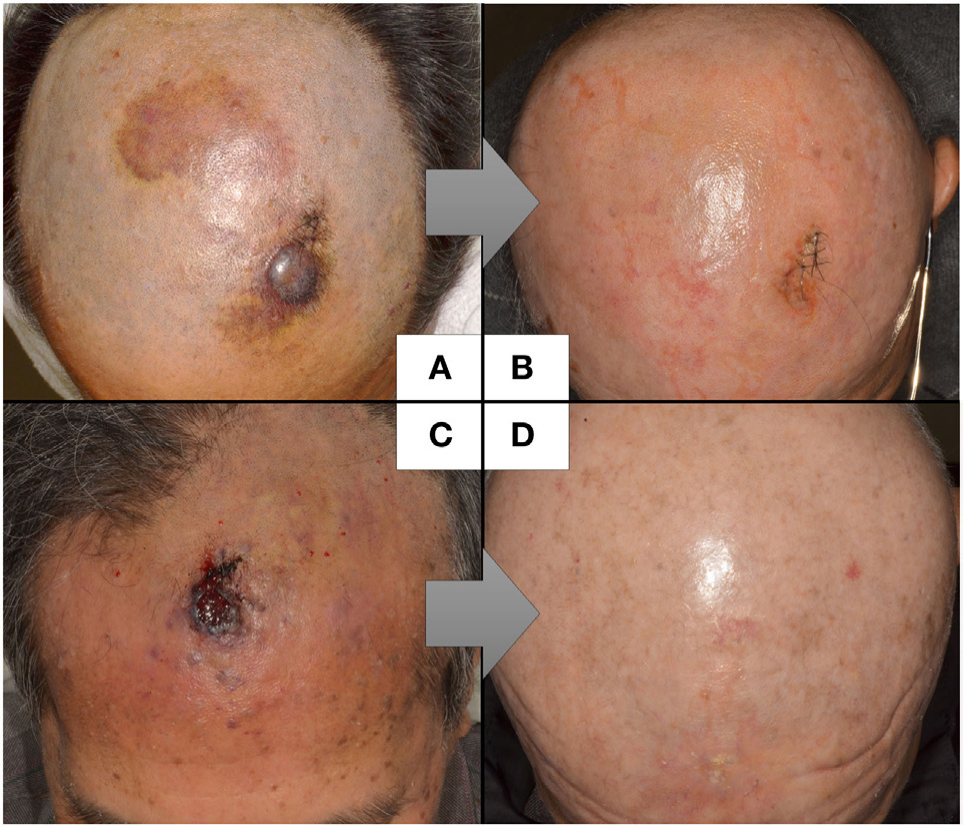
Representative cases of CAS treated by concurrent CRT.

The reported cases of CAS treated with CRT are described in Table 4. Because the study by Mark et al. (47) did not describe the timing of the chemotherapy, we could not determine whether they used chemotherapy concurrently or concomitantly with radiation. In the study by Miki et al. (98), 5 of the 12 patients who received docetaxel, the schedule was adjusted so that the drug was administered concurrently only on the first and last weeks of radiation. Another seven patients received docetaxel for 2–6 weeks during radiation in accordance with patient status. All the patients in the other two studies received chemotherapy and radiation concurrently (19, 98, 99). Most of the arms are composed of scalp CAS, which correlated with poor survival. The response to CRT was 82% (98) and 94% (19), with a statistically higher median overall survival than that of surgery followed by radiation in both studies. Representative photographs of patients who received CRT are presented in Figures 1A–D.

**Table 4:** Study using chemotherapy and radiation therapy for CAS.

Study	*N*	Patients tumor location/size	Treatment	RT dose	Response/pattern of failure	Median OS (months)
Mark et al. (47)	5	Scalp: 4 and Face: 1 Size not described	Anthracyclines	30–76.2 Gy	Response ratio: N.D. Local: 2/5 Distant: 2/5	27.0

	4	Scalp: 1 and Face: 3 Size not described	*Surgery*	50–65 Gy	Local: 1/4 Distant: 0/4	Not reached

Rhomberg et al. (99)	1	Scalp/face: 1 T2	Razoxane Vindesine	35–66 Gy	CR Alive without failure	41

Miki et al. (98)	11	Scalp: 16 and Face: 1 T1: 1 and T2: 15	Docetaxel	70 Gy	Response ratio: 82% Local: 4 Distant: 5 Both: 4	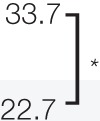
	5		*Surgery/IL-2*			
	
Our study (19)	16	Scalp: 14, and Extremity: 2 T1: 2 and T2: 14	Docetaxel	<65Gy:12 ≧65Gy:16	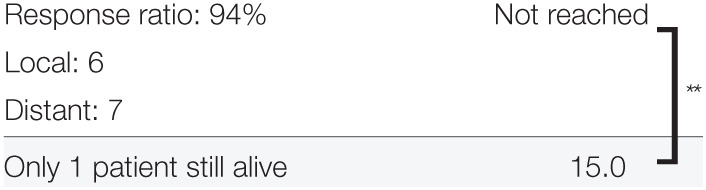	

	12	Scalp: 10, extremity: 2 T1: 2 and T2: 10	*Surgery*			

*P < 0.05.

**P < 0.01.

*OS, overall survival; CAS, cutaneous angiosarcoma; N.D., not described; IL-2: interleukin-2*.

Concurrent CRT brings severe side effects than when each treatment is delivered as monotherapy. In our study, 78% of the patients who received concurrent CRT had CTCAE grade-4 neutropenia, but the neutropenia was made manageable by use of granulocyte-colony stimulating factor and no treated-related death was observed (19). In the study by Miki et al. (98), all the patients developed grade 1–3 dermatitis but healed uneventfully.

Taking these finding together, CRT using taxanes could achieve satisfactory antitumor activity with good tolerability and might bring better survival than does conventional surgery followed by radiation especially for CAS of the scalp. Although the use of taxanes concurrently might bring severe side effects, we suggest concurrent CRT to gain maximum antitumor effect as long as the side effects are tolerable and manageable.

#### Maintenance Chemotherapy

To prevent locoregional and distant failure after response to chemotherapy, some previous report continued chemotherapy to maintain the response. Gambini et al. (100) achieved complete remission of radiation-induced angiosarcoma after treatment with paclitaxel and maintained the response for 4 years by maintenance therapy with intervals of no longer than 3 weeks. Interestingly, they had local recurrence twice when the treatment was delayed, but in both instances, a new complete remission was rapidly achieved with the same treatment and the patients remained disease-free at the time of their report. Nagano et al. (101) reported nine CAS patients treated with docetaxel, eight of whom continued docetaxel for 3–22 months (Table 5). None of the patients developed distant metastasis during maintenance chemotherapy. Rhomberg et al. (99) treated nine patients with angiosarcoma (five with thyroid, one with left ventricle, one with bladder, and one with scalp/face) with concurrent CRT using razoxane and vindesine. Complete remission of the tumor was obtained in six patients, five of whom received maintenance chemotherapy for 6 weeks to a year. Of those five patients, two developed recurrence but only one developed it during the maintenance chemotherapy.

**Table 5:** Maintenance chemotherapy after primary therapy.

Study	*N*	Tumor location	Primary chemotherapy	RT dose	Maintenance chemotherapy and duration (months)		Pattern of failure	Median OS (months)
Nagano et al., 2007 (101)	9	Scalp: 6, Face: 1 Neck: 1, Leg: 1	Docetaxel	–	Docetaxel	3–22 MD: 13.5	Local: 4 Distant: 0	Not described

Rhomberg et al. 2009 (99)	5	Thyroid: 4, Scalp/face: 1	Razoxane Vindesine	35–66 Gy MD: 56 Gy	Razoxane Vindesine	6 weeks-36 MD: 12	Local or distant: 2	27.0

Our study2014	9	Scalp: 7, Limb: 2	Docetaxel	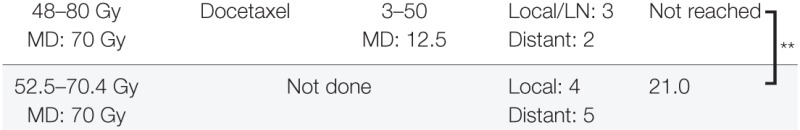				

	7	Scalp: 7						

** P < 0.01. *RT, radiotherapy; MD, median; OS, overall survival*.

In our study (19), 16 CAS patients were treated with concurrent CRT and 9 of them received maintenance chemotherapy. Locoregional relapse was seen in three of the nine patients who received maintenance chemotherapy, whereas it was seen in four of the seven patients who did not receive it. On the other hand, only two of the nine patients who received maintenance chemotherapy developed distant metastasis, whereas five of the seven patients who did not receive maintenance chemotherapy did develop distant metastasis (*P* < 0.05). A study by Ito et al. (75) showed that 19 patients who received maintenance chemotherapy using taxanes had significantly better survival than did 24 patients who received maintenance chemotherapy without taxanes (*P* < 0.0024) Collectively, maintenance chemotherapy after remission obtained by CRT seems to suppress tumor regrowth and development of distant metastasis. However, there is no consensus as to how long this maintenance chemotherapy should be continue. Further investigation is needed to determine the optimal length of maintenance chemotherapy.

#### Adjuvant/Neoadjuvant Chemotherapy

The use of adjuvant chemotherapy after complete removal of the tumor is attractive because we experience many CAS patient who develop distant metastasis even though there is no locoregional failure. However, anthracycline-based adjuvant chemotherapy did not show any survival benefit in soft tissue sarcomas (102). Indeed, we could not see any survival benefit in CAS patients by using taxanes after surgery and radiation (7). Similarly, adjuvant chemotherapy did not show a clear benefit among angiosarcoma patients treated with anthracyclines, paclitaxel, and other combinations (5, 6, 41, 44).

Some groups reported the use of chemotherapy before surgery (neoadjuvant chemotherapy) but did not show any survival benefit in face CAS (103) or in head and neck CAS (5). However, a certain percentage of patients who received neoadjuvant chemotherapy could achieve a complete response (60% in face CAS (103)) and did not require definitive surgery. Thus, the effect of neoadjuvant chemotherapy is difficult to interpret.

Since no large prospective study has been conducted to evaluate the value of adjuvant and neoadjuvant chemotherapy, those previous studies should be read with caution. However, the largest retrospective analysis of CAS including 821 patients indicated that both adjuvant and neoadjuvant therapy after surgery did not show any survival benefit on univariate and multivariate analyses (32). Further prospective study is required to evaluate the role of adjuvant/neoadjuvant chemotherapy for CAS.

### New Drugs

#### Anti-VEGF Drugs

Angiosarcomas express VEGFR (95, 97, 104), and overexpression of VEGF converted slow-growing vascular endothelial tumors to fast-growing malignant tumors in a mouse model and formed invasive angiosarcoma in immunodeficient mice (105). Conversely, blockade of the VEGF/VEGFR pathway inhibited tumor growth *in vitro* (106). Therefore, it is reasonable for the treatment to target the VEGF/VEGFR signaling pathway. Several studies using anti-VEGF monoclonal antibody (bevacizumab) have shown antitumor activity in angiosarcomas: 4 of 30 patients treated with bevacizumab had a partial response, with a mean time to progression of 26 weeks (107), and 2 of 2 patients treated with bevacizumab and radiation had a complete response (108).

On the basis of this background, Ray-Coquard et al. (74) conducted a non-comparative, open-label, randomized phase-2 trial to explore the activity and safety of bevacizumab and paclitaxel therapy for patients with advanced angiosarcoma. Fifty patients were randomized and assigned to two arms: (1) the paclitaxel alone or (2) the paclitaxel and bevacizumab arm. From the findings, they concluded that there is no benefit from adding bevacizumab to paclitaxel (median overall survival: 19.5 versus 15.9 months).

Other than monoclonal antibody, two small-molecule multi-tyrosine kinase inhibitors that can inhibit the VEGF/VEGFR signaling pathway have been used for the treatment of angiosarcoma patients: sorafenib (109) and pazopanib (110). A phase-2 trial including 37 patients with recurrent or metastatic angiosarcoma treated with sorafenib showed a response ratio of 14% with median progression-free survival of 3.8 months (111). No clinical trial to evaluate pazopanib activity in angiosarcoma has been conducted. In a case series using pazopanib for the treatment of taxane-resistant CAS, two of five patients achieved a partial response with median progression-free survival of 94 days (112). On the other hand, a case series of eight CAS patients treated with pazopanib did not show any benefit (113). Although we do not have enough conclusive evidence, the current first-line treatment should still be taxanes and anti-VEGF pathway therapy should be considered as the second- and third-line therapy.

#### Eribulin Mesylate

Eribulin mesylate suppresses microtubule polymerization and sequesters tubulin into nonfunctional aggregates, which is a mechanism distinct from those of other tubulin-targeting drugs such as taxanes (114). A phase-3 study comparing dacarbazine and eribulin in patients with advanced liposarcoma or leiomyosarcoma showed improved survival in patients treated with eribulin (115). This phase-3 study did not include angiosarcoma, and therefore, we do not have any evidence on the effect of eribulin for angiosarcoma. However, both taxanes and eribulin target microtubule polymerization, and eribulin binds to a different site of the microtubule (116), indicating that it may be effective for patients who become resistant to taxanes. Albeit in a case report, eribulin was shown to be effective for a patient who became resistant to docetaxel (117). Currently, we are conducting a prospective, observational clinical study to evaluate eribulin in patients with CAS who became resistant to taxanes (UMIN000023331); patient enrollment for this study is expected to be completed in 2018.

#### Checkpoint Inhibitors

Recent development of checkpoint inhibitors in melanoma treatment dramatically improved the survival of advanced melanoma. Melanoma with higher expression of programmed death receptor ligand-1 (PD-L1) correlated with a better treatment outcome when using anti-PD-1 antibody (118). This result supports the notion of a proposed immune escape mechanism by tumor cells using their PD-L1 expression on the cell surface to bind PD-1 on cytotoxic T cells and attenuate the immune response (119). Interestingly, our study group showed that CAS with PD-1 positive cell infiltration and tumor site PD-L1 expression correlated with survival (120). This result raises the possibility of using anti-PD-1 antibody for the treatment of CAS. To the best of our knowledge, there is no on-going or planned clinical trial to use checkpoint inhibitors for advanced angiosarcoma (clinicaltrials.gov).

## Current Recommendation and Future Perspective

The treatment of CAS, especially T2 tumors of the scalp, is still challenging. The surgical approach seems to be difficult because such tumors usually have an unclear border and often have skip lesions that make it difficult to determine the “true” tumor border. As patients with tumors larger than 10 cm were reported to have a catastrophic prognosis (35, 36), the current standard wide-margin resection followed by wide-field radiation might be palliative rather than curative (6). Radical surgery can reduce the tumor load; however, surgery-based treatment cannot target “subclinical” metastasis, which may have already occurred by the time of diagnosis. Therefore, we strongly recommend starting systemic chemotherapy along with primary tumor therapy. CRT can achieve this task: systemic administration of taxanes can target subclinical metastases and also act as a radiosensitizer that will enhance the effect of radiation therapy against the primary tumor. Although neoadjuvant chemotherapy and adjuvant chemotherapy may also achieve this task, to the best of our knowledge, no study has shown the superiority of this strategy.

Collectively, we suggest considering concurrent CRT using taxanes when we encounter CAS of the scalp with a T2 tumor. We also recommend maintenance chemotherapy even if complete remission of the tumor has been achieved. On the other hand, for T1 CAS with a clear tumor border, the current standard surgery followed by radiation might be sufficient to obtain a successful result. However, these recommendations are based on a small number of retrospective studies. CRT and maintenance chemotherapy should be evaluated with prospective clinical studies to confirm the superiority of this strategy.

Moreover, we currently do not have many options for when the tumor becomes resistant to taxanes. We have already launched a clinical study to evaluate eribulin mesylate as the second-line treatment after taxane-failure. Several clinical studies are now ongoing or planned to evaluate the effect of multi-kinase inhibitors such as sorafenib or pazopanib (clinicaltrials.gov). We hope the treatment of CAS will be dramatically improved, as it has for melanoma, in the near future.

## Author Contributions

I have full responsibility of this article. YF wrote the part of the manuscript. KY, TF, YN, NO, RW, YI, and MF confirmed the manuscript for submission.

## Conflict of Interest Statement

The authors declare that the research was conducted in the absence of any commercial or financial relationships that could be construed as a potential conflict of interest.
